# Atlanta Academy of Medicine

**Published:** 1877-12

**Authors:** J. S. Todd

**Affiliations:** Reporter


### Reports of Societies.

### ATLANTA ACADEMY OF MEDICINE.

J. S. TODD, M.D., Reporter.

ATLANTA, GA., November 13, 1877.

Dr. V. H. Taliaferro, President, in the chair.

Dr. Calhoun called attention to a case of epithelial cancer of the lids of the right eye, which was unusual, as that it had existed for thirty-eight years, the patient now being 67, stout and healthy, the mother of six or eight healthy children, none of whom exhibit any marks of the cancerous cachexia. For thirty-six years the cancer was dormant, though perceptible at the inner canthus of the right eye. Two years since it began to grow, but even twelve months ago it was not larger than a finger-nail, at which time he first saw her. It has gradually extended to the margin of the upper lid, along which it encroached, taking in some of the mucous membrane for a line and a half, and there were evidences of implication of the lachrymal bone. The operation, eight days ago, for its excision, was done under ether, of which she took two pints, the largest quantity he had given to any patient at one time. A cut was made from the bridge of the nose to half-way between the canthi. Found it adherent to the periosteum of the lachrymal bone, and shreds which he took to be cancerous dipping behind the globe of the eye. The cavity left after extirpation was large enough to contain his thumbs, leaving quite a deformity. This was rectified by a plastic operation. The patient being old, there was an abundance of loose skin, which permitted that the cut edge of the lid be drawn to the nose and there attached. The wound healed by the first intention, and all is now doing well—no fever or pain in the parts.

Dr. Baird reported an exceedingly interesting cure by arsenic of a facial neuralgia, that had existed for fifteen years. The subject was a gentleman æt. 35, who first caught malaria in Florida, and suffered from its effects during the war, while confined in a federal prison. His general health was shattered, his hopes gone. He had consulted many practitioners, and taken the usual amount of patent nostrums. The infra and supra-orbital and maxillary branches of the fifth pair of nerves were all affected. The pain was constant, at times, owing to spasmodic contractions, almost unsupportable. The patient found of late the greatest benefit from a series of successive small blisters, with which his face and head were dotted; on these blistered surfaces he sprinkled morphia. Dr. B., with many misgivings, commenced the treatment by using galvanism daily, which gave much immediate relief—resting the face, as the patient expressed it. He was also given, for a time, ten grains quinia daily, but this was soon discontinued. Fowler's solution of arsenic, in doses of four drops ter die, increased daily a drop until its physiological effects were produced—which generally happened in twenty days—was the main feature in the cure, which occurred in about two months. When arsenic caused puffiness, etc., it was discontinued for a few days, but pushed again and again, until success was procured. Six months have now elapsed, and there has been no return of the pain. Is of the opinion that neuralgia is frequently caused by malaria, and that arsenic will cure where quinine fails, even when given in full doses, say 30 or 40 grains ter die. He attempted to use the Fowler's solution hypodermically, but saw that abscess would result if persisted in.

Dr. Taliaferro was very much impressed with the effects of pressure on the uterus, by means of a tampon per vaginam, in relieving hypertrophic elongations of the neck of the womb, in cases of sub-involution. The facility with which pressure could be procured in this way, only needed a little thought and a study of the anatomy of the parts. Fill the vagina firmly, but not tightly or painfully, to the pubic bones, with sheep's wool soaked in glycerine, and on the female assuming the erect posture the superimposed contents of the pelvis press the womb steadily down, which, by causing fatty degeneration, results in absorption. In a recent case of elongation of the hypertrophied cervix, which he had determined to amputate, as a preparation for the latter procedure he filled the vagina with wool, and upon removing it, some time afterwards, saw so much improvement that the treatment was continued, and has resulted in reducing the cavity, in two weeks time, from six to three and a half inches in depth, and has, of course, obviated the section. He generally allows the packing to remain three days, but has left them for a week without detriment. The patient should always be placed in the knee and elbow position, this causing the vagina, when air is let in, to swell out as a balloon. Glycerine being detergent, should be preferably used to lubricate the wool—sheep's wool being preferable to the cotton on account of its elasticity, which prevents it from clogging together and becoming hard.

---

Atlanta, Ga., November 20, 1877.

Dr. J. S. Todd, esssayist for the month, read an interesting paper on belladonna.

Dr. J. G. Westmoreland desired to corroborate Dr. Todd's report of its effects in the relief of neuralgia. A case was presented for treatment several years ago, in the person of a lady about 22 years old, the subject of neuralgia of the scalp, which was not even temporarily relieved by full doses of opium. Quinine, also, failed to moderate the daily paroxysms. Belladonna was resorted to, and given in the dose of ten to fifteen drops of the tincture every five or six hours, so as to keep the pupils pretty fairly dilated. In thirty-six hours the pain entirely disappeared. Altogether, the action of the remedy was not kept up more than forty-eight hours, and complete relief was experienced for several years afterward.

Dr. J. M. Johnson said he had been accustomed to give belladonna in habitual constipation, along with strychnine and calomel, in the dose of a quarter of a grain of the extract to half a grain of calomel and the sixtieth of a grain of strychnine. He had also been in the habit of using belladonna for incontinence of urine, with success. With reference to its use in pulmonary phthisis, alluded to in the very able paper of Dr. Todd, just read, he could not, from experience, give any opinion; but from the opinion of its physiological action, given by Dr. Williams, of England, and the pathology of tuberculosis, as given by the same author, it would seem to afford a rational plan of treatment for that disease. He also alluded to a case of atresia of the cervix uteri, with painful menstruation, in which half a grain of the extract of belladonna was given three times a day for two days, with complete relief, but not without some unpleasant symptoms, temporarily, on account of the large dose used.

Dr. Taliaferro referred, approvingly, to that portion of Dr. Todd's essay condemning the prevailing mania for new remedies, in place of investigating and understanding more perfectly the physiological action and therapeutic effects of old and familiar remedies. He was in favor of enquiring more particularly into the manner of action by which the various beneficial results were had. He had some idea of the peculiar impression made on the capillaries by belladonna, from which some of the therapeutic effects, alluded to in the essay, may result.

Dr. J. G. Westmoreland agreed with Dr. Taliaferro as to the importance of studying more closely the direct or physiological action of remedies, in order to their rational use, and thought the able and interesting paper of Dr. Todd would be more perfect by considering belladonna in this respect.

Dr. Todd mentioned a case of delivery, recently attended, in which post partem hemorrhage proved troublesome, and which was arrested by the introduction of the hand into the uterus, the use of ergot, cold water and ice. He desired the opinion of members as to the probability of further difficulty in the case from the hemorrhage which occurred, or the treatment instituted for its relief.

Dr. J. M. Johnson, in reply, expressed the opinion that the manipulation necessary to arrest violent hemorrhage, probably leaves the womb more subject to inflammation, etc., but that such consequences do not generally follow. He mentioned a case in which great difficulty was found in arresting the flow of blood—no unpleasant effects resulting from the rough manipulation found necessary.

Dr. J. G. Westmoreland thought no serious consequences need be apprehended, necessarily, from the accident, nor the means instituted for its arrest.

Dr. Taliaferro thought that while metritis and other difficulty delaying the recovery may be more likely to occur, yet that he should feel no fear in this case.

Dr. J. M. Johnson reported two cases of impotence, treated during last summer, with Squibb's fluid extract of ergot. One, the subject of malarial poison, had been impotent for twelve months, probably on account of general depraved condition of the system, from the unhealthy locality in which he lived. One teaspoonful of the fluid extract, three times a day for a week, restored virility perfectly. The other case had symptoms indicating irritation of the medulla oblongata, for which he had been treated unsuccessfully by counter-irritants, etc. This case was treated in the same manner as the first mentioned, and in three days was restored to normal activity of the procreative organs.

Dr. Baird thought it unreasonable to suppose that ergot is a panacea for all cases of impotence, since the condition is not always the result of the same cause. When it depends upon congestion of the spinal cord, he could very well understand why ergot would be useful; for the action of this remedy upon the cord tends to relieve this condition. Impotence is sometimes the result of urethral stricture, and other causes, which ergot cannot possibly relieve, and, therefore, cannot be given in all cases of impotence with any prospect of success.

---

Atlanta, Ga., November 26, 1877.

Academy met, Dr. V. H. Taliaferro, President, in the chair.

Dr. Todd, referring to the case of post partem hemorrhage, reported at the last meeting, remarked that no further difficulty had been met with in the case since the hemorrhage was arrested.

Dr. Taliaferro reported a case of pelvic peritonitis in a lady, recently the subject of opium habit. She had advanced so far in the use of opium that fifteen grains of morphine were used daily, when the cure was commenced by gradual reduction. One month since, the habit was entirely suspended, after having been under treatment for about three months. About three days ago she was attacked with inflammation of the pelvic viscera, as above stated, from which she suffered constant excrutiating pain, rendering her unable to sleep or rest in any manner. Knowing the short time that had elapsed since she had been relieved from the habit of taking a large quantity of opium, he supposed that more than the ordinary quantity would be required to give her ease, and commenced with the dose of half a grain of morphine. This giving no relief from the suffering, the dose was increased to one grain every hour, and finally to two grains, hyperdomically, every two hours, with like failure. Frequent discharges from the bowels coming on, the preparation and mode of administration were changed to laudanum, in the dose of half a teaspoonful by the stomach, and a fluidrachm by the rectum. No relief being afforded, the black drop was then used, in the dose of two fluidrachms every two hours, which in a few hours was followed by perfect relief from pain. He also called attention to a novel remedy for indigestion, which he had met with in domestic practice. In conversation with an intelligent lady patient who had been absent from the city, and had been a slight sufferer from indigestion, he learned that relief had been afforded by sand obtained through a friend from a spring in the neighborhood. The conclusion arrived at, therefore, was that sharp sand obtained anywhere, is equally effectual. Obtaining a specimen of the sand from his informant, Dr. T. tested its effects upon himself and others laboring under symptoms of indigestion, with rather promising results; and finding nothing different from other sharp, clean sand, does not think it necessary to obtain the article from any particular source. He uses it in the dose of one-third to half a teaspoonful, and called upon Dr. Simpson for the report of the effects of that given him in a state of embarrassed digestion.

Dr. Simpson reported rather negatively, saying he had only taken one dose, and although the symptoms disappeared not long afterwards, he could not certainly determine what agency the sand exercised.

Dr. J. G. Westmoreland thought it not unreasonable that sand should exert mechanical influence upon the surface of the stomach, such as is had by coarsely pulverized charcoal, bran, alone or in bread made of unbolted flour. These rough substances have for a long time been recognized by the profession as suitable means to promote digestion in sluggish condition of the stomach. The explanation is that the mucous surface is titilated, and thereby excited. This being communicated to the whole organ, peristaltic contraction is increased, and perhaps the secretion of gastric fluid is promcted. It is reasonable to suppose that sand will act more certainly and powerfully in producing these effects upon the stomach through mechanical process than bran. It must be remembered, however, that indigestion is the result of very different, and even opposite conditions of the stomach. Instead of torpor, we often meet with excessive excitement, amounting to an inflammatory condition of the mucous surface, as the cause of indigestion. In such condition bran, sand, charcoal, or any other exciting agent, will not only fail to be useful, but must necessarily prove injurious.

Dr. Calhoun reported a case of cystic tumor at the corneo-sclerotic junction, coming on in connection with pterygium. It had been growing about twelve months and had attained the size of the little finger's end. He operated by grasping the tumor with forceps, and shaving it out, with the existing pterygium, leaving a depression in the cornea, where the base and larger part of the tumor was situated. Such tumors are rare, as in the great number of diseased eyes which he has seen, he had never met with more than two before.

He also referred to a case reported some year or two since, in which he extirpated a tumor, without removing the ball, taking its origin on the periosteum, around the optic foramin. It was thought most likely to be malignant, and had practically destroyed the vision of the eye involved. He recalled the case now, to say that a letter received from the subject recently, informs him that there has been no return of the growth, and that the ball and lid are almost natural, and the sight is nearly as perfect as in the other eye.

Adjourned.

